# Imprecision in tuberculosis infection outcomes: implications for non-inferiority vaccine trials

**DOI:** 10.1093/ije/dyag034

**Published:** 2026-03-04

**Authors:** Daniel J Grint, Richard G White, Gavin Churchyard, Andrew Fiore-Gartland, Molebogeng Rangaka, Alberto L Garcia-Basteiro, Frank Cobelens

**Affiliations:** Department of Infectious Disease Epidemiology, London School of Hygiene and Tropical Medicine, London, United Kingdom; Department of Infectious Disease Epidemiology, London School of Hygiene and Tropical Medicine, London, United Kingdom; Aurum Institute, Parktown, Johannesburg, South Africa; School of Public Health, University of Witwatersrand, Parktown, Johannesburg, South Africa; Department of Medicine, Vanderbilt University Medical Center, Nashville, TN, United States; Vaccine and Infectious Disease Division, Fred Hutchinson Cancer Center, Seattle, WA, United States; Faculty of Population Health Sciences, Institute for Global Health, UCL, London, United Kingdom; ISGlobal, Hospital Clínic—Universitat de Barcelona, Barcelona, Spain; Centro de Investigação em Saúde de Manhiça (CISM), Maputo, Mozambique; Centro de Investigación Biomédica en Red de Enfermedades Infecciosas (CIBERINFEC), Barcelona, Spain; Department of Global Health, Amsterdam Institute for Global Health and Development, Amsterdam UMC, Location University of Amsterdam, Amsterdam, the Netherlands; Amsterdam Public Health, Amsterdam, The Netherlands

**Keywords:** simulation study, type I error, power, interferon-gamma release assay

## Abstract

**Background:**

Randomized trials comparing new vaccines against tuberculosis for use in neonates and infants, for whom Bacille Calmette–Guérin vaccination is established practice, are using tuberculosis infection as the primary endpoint in a non-inferiority design. Markers of tuberculosis infection have imperfect sensitivity and specificity. Flaws in the non-inferiority trial design typically bias towards non-inferiority, which may result in falsely declaring non-inferiority.

**Methods:**

We conducted a statistical simulation study to assess the impact of imperfect markers of tuberculosis infection on the interpretation of tuberculosis vaccine trials testing a non-inferiority hypothesis of an infection primary outcome in a two-arm randomized comparison. Data were generated in three 2-year cumulative risk of tuberculosis infection scenarios (2%, 5%, and 8%). The specificity of tests of tuberculosis infection was assumed to range from 100% to 85%, while the sensitivity was assumed to range from 100% to 64%. Log-binomial regression was used to estimate the relative risk of tuberculosis infection.

**Results:**

With 100% sensitivity and specificity, type I error and power were both approximately equal to the expected values (2.5% and 80%, respectively) in all three cumulative tuberculosis risk scenarios. With modest deviations from perfect sensitivity and specificity (95% for both), the risk of falsely declaring non-inferiority was 96.8%, 53.2%, and 27.8% in the 2%, 5%, and 8% cumulative tuberculosis risk infection scenarios, respectively.

**Discussion:**

Tuberculosis vaccine non-inferiority trials using an infection primary outcome must be designed and interpreted accounting for the specificity of the tools used to measure infection, otherwise they risk declaring non-inferiority by default.

Key Messages

- We conducted a statistical simulation study to assess the impact of imperfect sensitivity and specificity in the primary outcome definition of tuberculosis infection in vaccine trials testing a non-inferiority hypothesis.
- With only modest departures from perfect specificity in tuberculosis infection markers, the risk of falsely declaring non-inferiority is substantial.
- Vaccine trials testing a non-inferiority hypothesis with an infection primary outcome must account for the imprecision in the tools used to define the outcome; otherwise, vaccines may be falsely declared non-inferior.

## Introduction

Non-inferiority trial designs are commonly used in the assessment of new treatments and other interventions where there is an established therapeutic standard of care and are also becoming increasingly common for trials of new vaccines [1–3].

Tuberculosis (TB) (*Mycobacterium tuberculosis*) is the most common cause of death due to a single infectious agent globally [4]. The only available TB vaccine, Bacille Calmette–Guérin (BCG), has existed for over 100 years and provides 80% (95% confidence interval, 34%–94%) protection against severe forms of TB disease in children but inconsistent protection against pulmonary TB at later ages [5]. The World Health Organization (WHO) has identified new vaccines against TB as a global health priority, and several candidates are in clinical development [6].

Presently, there are several ongoing randomized trials comparing new vaccines against TB for use in neonates and infants, for whom BCG vaccination is an established practice [6]. The WHO’s Product Profile Characteristics for a new TB vaccine for use in this age group stipulates that it should have superior efficacy for prevention of TB disease as compared to BCG, improved safety in neonates and infants with innate or acquired immunodeficiency, including HIV infected infants, or improved manufacturing scalability and lower costs [7].

In addition, TB disease has a low background incidence and long incubation periods, making trials with a prevention of disease outcome large, long, and costly. As TB infection occurs much more commonly than the disease, trials have sought to adopt prevention of TB infection as a surrogate primary outcome endpoint [8, 9]. Infection is generally measured as interferon-gamma release assays (IGRA) conversion that measures an adaptive immune response to *M. tuberculosis*-specific antigens. Although there is no gold standard for establishing TB infection, false-negative IGRA results can occur in people with bacteriologically confirmed TB [10]. False-positive IGRA results may occur with some non-tuberculous mycobacterial infections such as *Mycobacterium marinum* [11], but also because TB infections defined by lower IGRA cut-off values are known to progress to TB disease less often than TB infection based on higher IGRA cut-off values [12].

Some current trials are using a non-inferiority trial design in comparing the efficacy of new vaccines to that of BCG with TB infection as the primary endpoint. Non-inferiority trials pose a risk of falsely declaring non-inferiority under certain conditions relating to trial conduct and the choice of primary outcome [13]. This is because in a non-inferiority trial, flaws in trial design typically bias towards non-inferiority. This is a well-established principle for drug trials, where regulators provide extensive guidance on endpoint selection and trial management [14]. The same principles have not yet been clearly established for vaccine trials, which differ from drug trials in that the primary outcome is usually specified as a relative risk rather than a risk difference, which is interpreted as protective efficacy [15]. In vaccine studies, a relative risk is preferred because, contrary to a risk difference, it provides a measure of effect that is independent of the underlying disease incidence.

**Figure 1:**
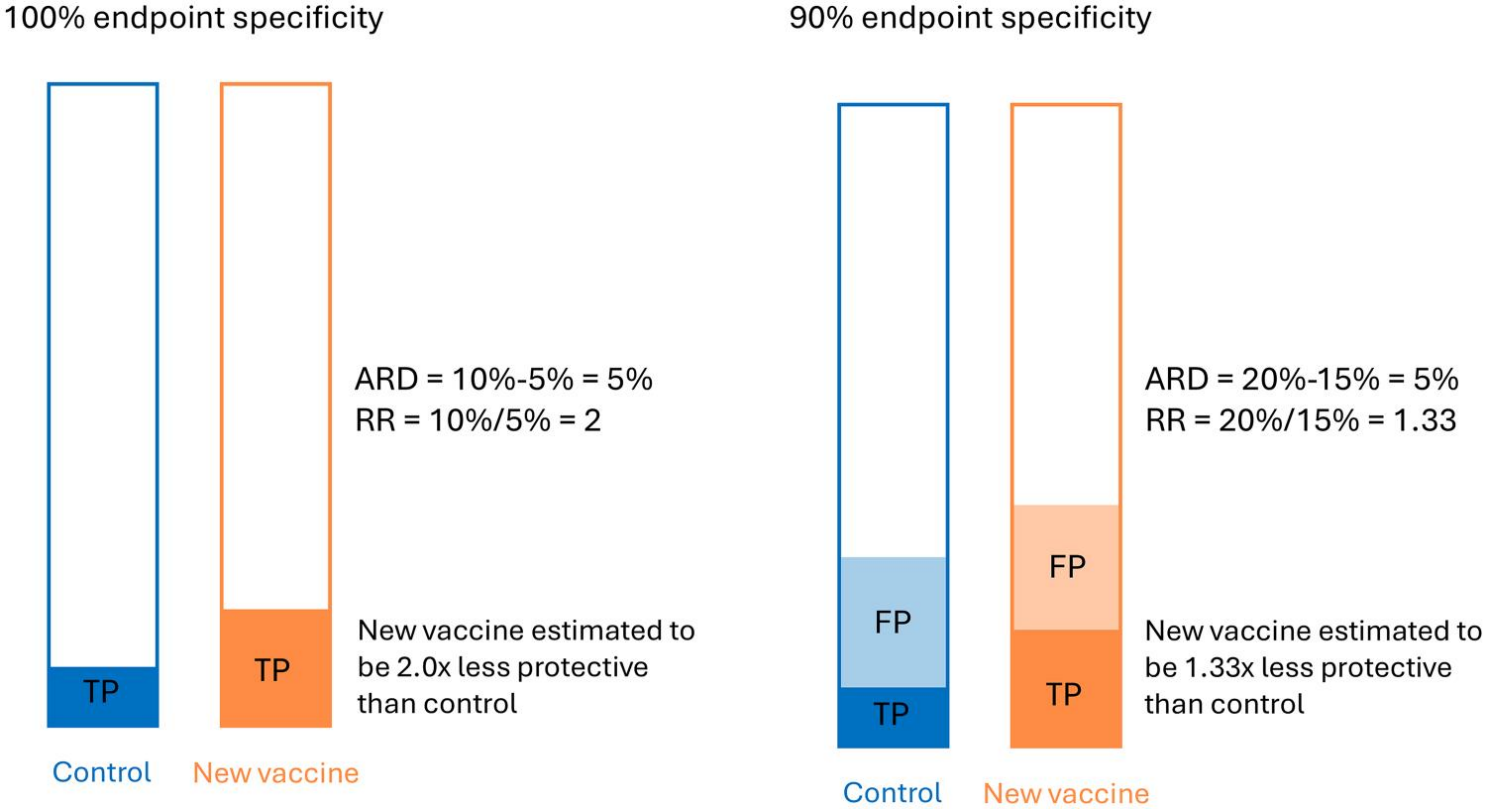
Hypothetical example showing the effect of incomplete specificity of the measurement of the endpoint in a vaccine trial on the estimated absolute risk difference (ARD) and estimated relative risk (RR). Bars represent the full study population for each trial arm, shaded areas the proportion positive on a test. The RR is biased towards 1, whereas the ARD is not affected. The vaccine’s efficacy is commonly interpreted as 1 − RR. FP, false positives; TP, true positives.

Since IGRA has suboptimal diagnostic accuracy for TB infection [10, 12], TB infection outcomes reported by trials may be dominated by false test positives rather than true TB infections, particularly in low infection incidence populations. As vaccine trials are typically analysed using relative effect measures, false test positive outcomes may diminish the true relative difference between control and experimental vaccines and result in falsely declaring non-inferiority (Fig. 1). Conversely, false test negative outcomes may diminish the statistical power for relative effect measures in a non-inferiority vaccine trial [16].

This simulation study assessed the impact of imperfect sensitivity and specificity of TB infection diagnostics on type I error and power in simulated non-inferiority trials.

## Materials and methods

We conducted a statistical simulation study to assess the impact of imperfect sensitivity and specificity in the outcome definition of TB infection in vaccine trials testing a non-inferiority hypothesis. We aimed to demonstrate the impact of imperfect outcome sensitivity and specificity on the type I error and power of non-inferiority vaccine trials.

Data were generated from the binomial distribution with probability equal to the assumed risk of TB infection following vaccination. Data were generated from hypothetical non-inferiority trials under three scenarios for the risk of TB infection in the control arm following vaccination, reflecting three different levels of cumulative incidence of IGRA conversion during 2 years of follow-up (2%—e.g. rural East Africa, 5%—e.g. urban East Africa, 8%—e.g. South Africa) [17].

The simulated sample size for each scenario was informed by standard statistical formulae based on the normal distribution approximation [18, 19], and refined in simulations to have approximately 80% power for the non-inferiority hypothesis, assessed at the upper confidence interval for the relative risk at a non-inferiority margin of 1.25, assuming a one-sided type I error probability of 2.5% (Table 1). We selected this non-inferiority margin assuming that a vaccine 1.25 times less efficacious than BCG in protecting neonates and infants against TB infection would not be considered non-inferior regardless of possible advantages with regard to safety or cost.

**Table 1:** Parameters for data generation.

			Power analysis		Type I error analysis	
Tuberculosis infection risk	Total sample size	Non-inferiority margin	Relative risk	Power	Relative risk	Type I error (one-sided)
0.02	32 000	1.25	1	80%	1.25	2.5%
0.05	12 000	1.25	1	80%	1.25	2.5%
0.08	7200	1.25	1	80%	1.25	2.5%

The trial populations were generated with 2000 replications to give an approximate Monte Carlo standard error of 1 percentage point for the estimated type I error and power [20].

Following data generation of the trial populations and ‘true’ TB infection cases, ‘observed’ TB infections were computed from a binomial distribution applying varying degrees of test sensitivity and specificity.

The targets of the analysis were the type I error and power of the test of the non-inferiority hypothesis of the experimental vaccine compared to the control. For both targets, the method of analysis was a log-binomial regression model computing the relative risk of TB infection.

For analysis of type I error, data were generated under a ‘true’ relative risk of 1.25. The type I error probability was computed as the proportion of simulated trials where the upper limit of the confidence interval for the relative risk comparing the experimental vaccine to the control vaccine was <1.25. In other words, type I error probability was calculated as the proportion of simulated trials where non-inferiority was demonstrated, when the true risk of TB infection was 1.25 times higher with the experimental vaccine.

For analysis of power, data were generated under a ‘true’ relative risk of 1. Power was computed as the proportion of simulated trials where the upper limit of the confidence interval for the relative risk comparing the experimental vaccine to the control vaccine was ≤1.25. In other words, power was calculated as the proportion of simulated trials where non-inferiority was demonstrated, when there was truly no difference in the efficacy of the experimental and control vaccines.

Analyses were conducted under the following assumptions on the diagnostic accuracy of the TB infection: sensitivity and specificity both 100%, sensitivity 100% and specificity 95%, sensitivity 95% and specificity 100%, sensitivity and specificity both 95%, and sensitivity 95% and specificity 98% (Table 2) [12, 21]. As an extreme case, we also included sensitivity 64% and specificity 85%. These values were calculated from a meta-analysis of cohort studies from low-incidence countries that included immigrants from high-incidence countries [10].

**Table 2:** Sensitivity and specificity combinations of tuberculosis infection markers used in data generation.

Sensitivity	Specificity
100%	100%
100%	95%
95%	100%
95%	95%
95%	98%
64%	85%

Data generation and statistical analysis were performed using R version 4.3.3 and R Studio 2023.12.0 build 369. The code used to generate the data and perform all analyses is available at https://github.com/dgrint/TB-Vacc.

## Results

### Analysis of type I error

Type I error probability here refers to the proportion of simulated trials where non-inferiority was demonstrated, when the true risk of TB infection was 25% higher with the experimental vaccine than the control.

**Figure 2:**
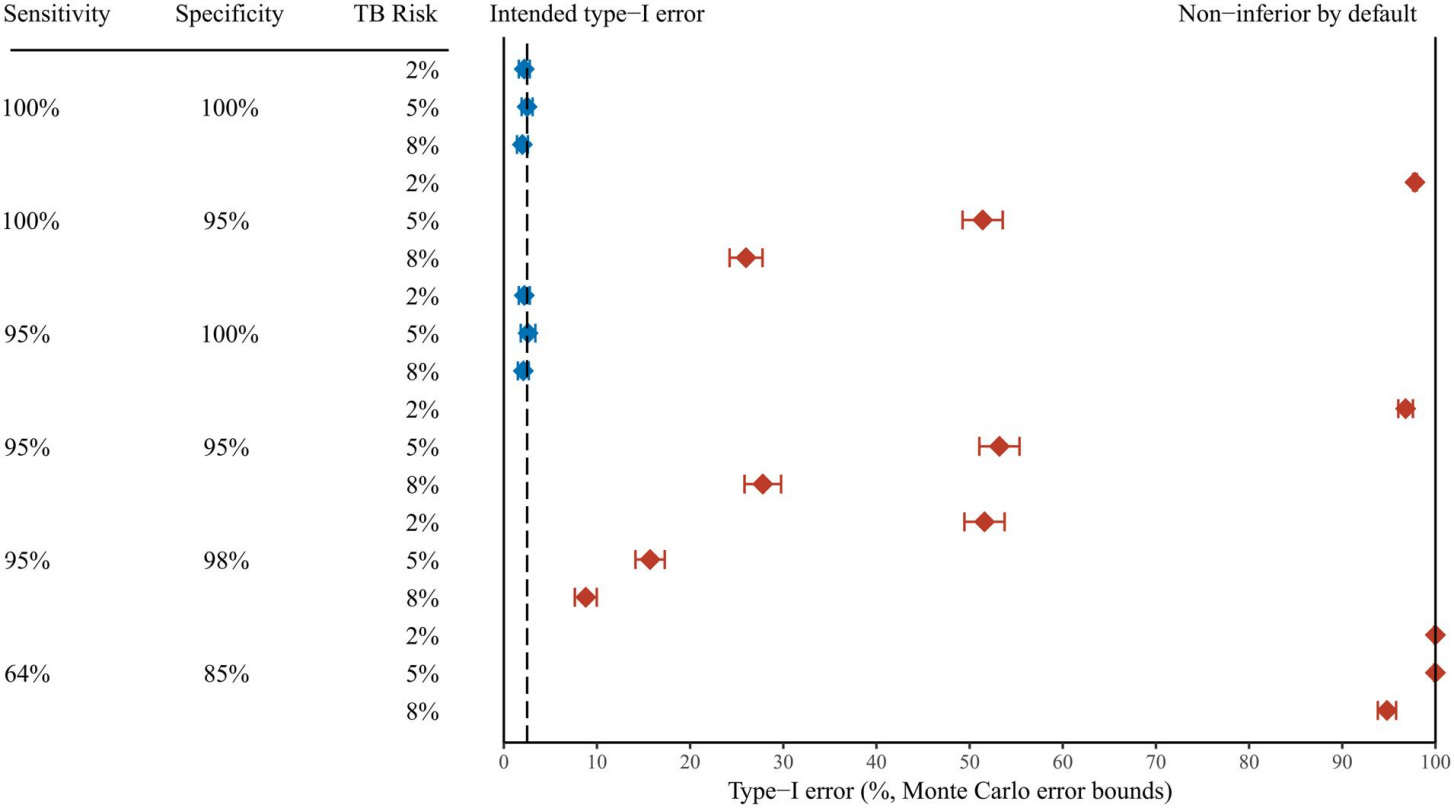
Probability of falsely declaring non-inferiority (type I error) for different combinations of sensitivity and specificity of the measurement of the endpoint, and the cumulative infection incidence, in 2000 simulated tuberculosis vaccine trials with an infection endpoint for which true risk is 25% higher with the experimental vaccine than with the control. Horizontal bars: 95% Monte Carlo error bounds. Blue diamonds: combinations for which the observed type I error is consistent with 2.5% (intended type I error). Red diamonds: combinations for which the observed type I error is larger than 2.5%. At 100% (right margin) non-inferiority will be declared by default (in all trials).

Results of the simulated type I error analysis are summarized in Fig. 2 and Table 3. When the sensitivity and specificity of the diagnostic tools are both 100%, the type I error probability is approximately equal to 2.5% for all three true TB infection risk scenarios.

**Table 3:** Impact of imperfect sensitivity and specificity of tuberculosis infection outcome on type I error.

	Control vaccine		Experimental vaccine		Type I error (alpha)		
Sensitivity; specificity	True risk of TB infection	Observed risk of TB infection	True risk of TB infection	Observed risk of TB infection	Expected type I error	Estimated type I error	Monte Carlo error
100%; 100%	0.02	0.02	0.025	0.025	2.5%	2.2%	0.3%
100%; 100%	0.05	0.05	0.0625	0.0625	2.5%	2.5%	0.3%
100%; 100%	0.08	0.08	0.10	0.10	2.5%	2.0%	0.3%
100%; 95%	0.02	0.069	0.025	0.074	2.5%	97.8%	0.3%
100%; 95%	0.05	0.0976	0.0625	0.109	2.5%	51.4%	1.1%
100%; 95%	0.08	0.126	0.10	0.146	2.5%	26.0%	0.9%
95%; 100%	0.02	0.019	0.025	0.024	2.5%	2.2%	0.3%
95%; 100%	0.05	0.0476	0.0625	0.0594	2.5%	2.6%	0.4%
95%; 100%	0.08	0.076	0.10	0.095	2.5%	2.1%	0.3%
95%; 95%	0.02	0.068	0.025	0.073	2.5%	96.8%	0.4%
95%; 95%	0.05	0.095	0.0625	0.106	2.5%	53.2%	1.1%
95%; 95%	0.08	0.122	0.10	0.140	2.5%	27.8%	1.0%
95%; 98%	0.02	0.039	0.025	0.043	2.5%	51.6%	1.1%
95%; 98%	0.05	0.0666	0.0625	0.0782	2.5%	15.7%	0.8%
95%; 98%	0.08	0.094	0.10	0.113	2.5%	8.8%	0.6%
64%; 85%	0.02	0.16	0.025	0.162	2.5%	100%	0.07%
64%; 85%	0.05	0.174	0.0625	0.181	2.5%	100%	0.07%
64%; 85%	0.08	0.189	0.10	0.199	2.5%	94.8%	0.5%

Type I error: probability of falsely declaring non-inferiority when the true risk of TB infection is 1.25 times higher with the experimental vaccine.

With 100% sensitivity and 95% specificity, the type I error probability increases to 97.8% when the true TB infection risk is 2%. Meaning non-inferiority would be falsely declared in 97.8% of trials where the true risk of TB infection was 1.25 times higher with the experimental vaccine. The type I error probability is 51.4% and 26.0% for true TB infection risks of 5% and 8%, respectively, with similar results when sensitivity and specificity are both 95% (96.8%, 53.2%, and 27.8%, respectively).

Sensitivity of 95% combined with perfect specificity had only a modest impact on the type I error probability. However, even a modest reduction in specificity to 98% resulted in inflated type I error probability for all three scenarios (51.6%, 15.7%, and 8.8%, respectively).

In the extreme case of 64% sensitivity and 85% specificity, type I error probability was inflated to 100% for the 2% and 5% TB infection risk scenarios and 94.8% for the 8% scenario. Meaning virtually all trials would demonstrate non-inferiority in this scenario.

### Analysis of power

Power here refers to the proportion of simulated trials where non-inferiority was demonstrated, when the true risk of TB infection was the same with the experimental vaccine and the control. Greatly inflated power indicates an overwhelming likelihood of demonstrating non-inferiority.

**Figure 3:**
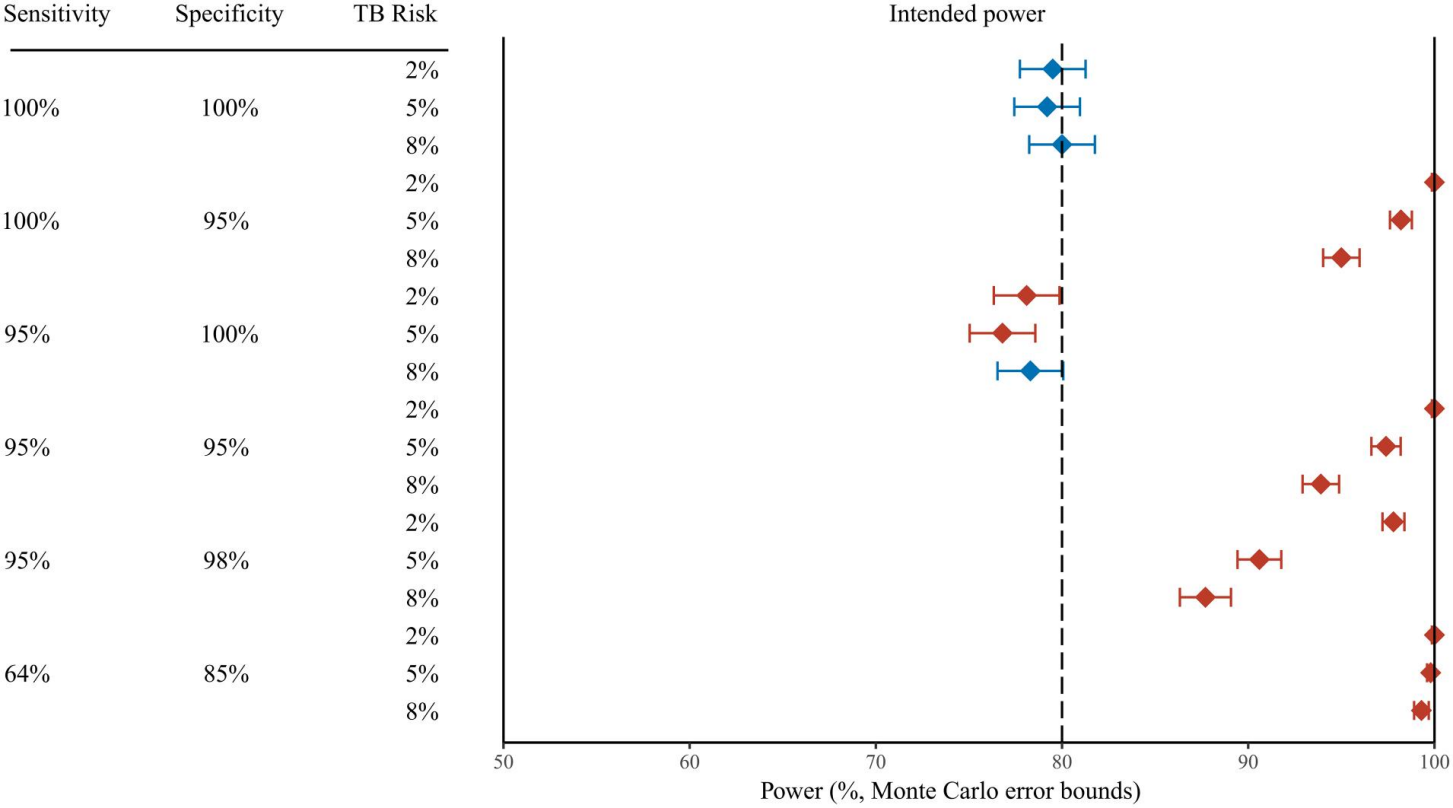
Probability of correctly declaring non-inferiority (power) for different combinations of sensitivity and specificity of the measurement of the endpoint, and the cumulative infection incidence, in 2000 simulated tuberculosis vaccine trials with an infection endpoint for which true risk is 25% higher with the experimental vaccine than with the control. Horizontal bars: 95% Monte Carlo error bounds. Blue diamonds: combinations for which the observed power is consistent with 80% (intended power). Red diamonds: combinations for which the observed power deviates from 80%. At 100% (right margin) non-inferiority will be declared by default.

Results of the simulated power analysis are summarized in Fig. 3 and Table 4. When the sensitivity and specificity of the diagnostic tools are both 100%, the observed risk of TB infection matches the true risk of TB infection, consequently, power is approximately equal to 80% for all three true TB infection risk scenarios.

**Table 4:** Impact of imperfect sensitivity and specificity of tuberculosis infection outcome on power.

	Control vaccine		Experimental vaccine		Power		
Sensitivity; specificity	True risk of TB infection	Observed risk of TB infection	True risk of TB infection	Observed risk of TB infection	Expected power	Estimated power	Monte Carlo error
100%; 100%	0.02	0.02	0.02	0.02	80%	79.5%	0.9%
100%; 100%	0.05	0.05	0.05	0.05	80%	79.2%	0.9%
100%; 100%	0.08	0.08	0.08	0.08	80%	80.0%	0.9%
100%; 95%	0.02	0.069	0.02	0.069	80%	100%	0.07%
100%; 95%	0.05	0.0976	0.05	0.0975	80%	98.2%	0.3%
100%; 95%	0.08	0.126	0.08	0.126	80%	95.0%	0.5%
95%; 100%	0.02	0.019	0.02	0.019	80%	78.1%	0.9%
95%; 100%	0.05	0.0476	0.05	0.0475	80%	76.8%	0.9%
95%; 100%	0.08	0.076	0.08	0.076	80%	78.3%	0.9%
95%; 95%	0.02	0.068	0.02	0.068	80%	100%	0.07%
95%; 95%	0.05	0.095	0.05	0.095	80%	97.4%	0.4%
95%; 95%	0.08	0.122	0.08	0.122	80%	93.9%	0.5%
95%; 98%	0.02	0.039	0.02	0.039	80%	97.8%	0.3%
95%; 98%	0.05	0.0665	0.05	0.0665	80%	90.6%	0.6%
95%; 98%	0.08	0.0945	0.08	0.0942	80%	87.7%	0.7%
64%; 85%	0.02	0.16	0.02	0.16	80%	100%	0.07%
64%; 85%	0.05	0.174	0.05	0.175	80%	99.8%	0.1%
64%; 85%	0.08	0.189	0.08	0.189	80%	99.3%	0.2%

Power: Probability of declaring non-inferiority when the true risk of TB infection is the same for the control and experimental vaccine.

When the specificity is below 100%, the observed risk of TB infection is considerably higher than the true risk of TB infection. When 1 − specificity is greater than the true risk of TB infection, there are more false positives than true positives in the observed risk of TB infection.

With 100% sensitivity and 95% specificity, power is ≥95% in all scenarios. With 95% sensitivity and 95% specificity, power remains ≥93% for all scenarios, indicating that imperfect sensitivity does not counterbalance imperfect specificity.

Sensitivity of 95% combined with perfect specificity has a modest impact on power, reducing it below 80% in all three scenarios. However, even a modest reduction in specificity to 98% results in inflated power for all three scenarios, and considerably so when the true risk of TB infection is low.

In the extreme case of 64% sensitivity and 85% specificity, power is inflated to approximately 100% for all three TB infection risk scenarios.

## Discussion

This study demonstrated that even modest departures from perfect specificity in tests of TB infection outcomes result in vastly inflated type I error and power in trials using TB infection as the primary outcome. Consequently, the risk of declaring non-inferiority by default is high, particularly when the true risk of TB infection over the follow-up period, in this case 2 years, is similar or less than the 1 − specificity of the diagnostic test.

This study assessed the impact of modest, as well as extreme, departures from perfect diagnostic performance. There is no established gold standard diagnostic test to define TB infection. The sensitivity of IGRA has generally been assessed among patients with bacteriologically confirmed TB and estimated at close to 95% for the latest generation assays [21]. Various approaches have been used to assess the specificity of IGRA for TB infection for use in vaccine trials. The estimate that matters most is that for predicting TB disease occurring over a 2-year period [22].

A cohort study that followed South African infants in a high TB incidence setting 6–24 months post QuantiFERON testing showed close to 95% specificity for TB disease at the manufacturer-recommended cut-off of 0.35 IU/ml and close to 98% specificity at a cut-off of 4.0 IU/ml [12]. However, the specificity calculated for any IGRA test at manufacturer-recommended cut-off from a meta-analysis of cohort studies from (mainly) low-incidence countries that included immigrants from high-incidence countries of all ages was only 85% [10]. The difference may be due to differences in age groups, which would make the higher specificity estimate more applicable for our study. It may also reflect technical variations in performing the test in field conditions and differences in the incidence of TB infection. In multi-country TB vaccine trials, IGRA may therefore have a specificity closer to our extreme case of 85%. The sensitivity calculated from the meta-analysis of cohort studies was 64% [10]. Although this low estimate may partially reflect bias due to infections occurring between IGRA testing and diagnosis of TB disease [22], we included it in our simulations as an extreme case.

The probability of falsely declaring non-inferiority was dependent on the cumulative risk of TB infection in the population where the trial was conducted. Most TB vaccine trials will be conducted in populations with 5% to 8% cumulative infection risk over 2 years trial follow-up. Our simulations suggest that the probability of falsely declaring non-inferiority was lowest for the highest incidence setting, but still inflated 10-fold (26%) when specificity was 95%. In the extreme case of 85% specificity, the probability of falsely declaring non-inferiority was 94.8% even at the highest background incidence of TB infection.

We assumed that TB infection status was measured only once, at the end of trial follow-up. In reality, trials may do repeated IGRA testing, e.g. at 6-month intervals. In such cases, false-positive IGRA results will occur at every test round, and the overall specificity may be even lower, further increasing the risk of falsely declaring non-inferiority. On the other hand, if sustained infection, requiring two or more serial positive tests, is a requirement to determine infection, the specificity of the endpoint may increase [9].

It could be argued that the results presented here are not surprising, as the observed TB infection risk is different from the true TB infection risk that was used to inform the sample size calculations. The analysis performed on the observed risk of TB infection is then a departure from the study design, leading to the inflated type I error and power shown here. Indeed, researchers must account for the precision of the tools measuring their primary outcomes when designing their trials. However, when the proportion of false-positive results due to the diagnostic tool is close to or higher than the risk of the disease itself, the validity of the tool as a measure of defining the primary outcome must be called into question.

The considerations underlying our analyses may also have implications for non-inferiority trials with TB disease as the endpoint. Trials among neonates and infants in whom bacteriological confirmation of the diagnosis can be challenging often have composite clinical endpoints; therefore, diagnostic specificity of the outcome in these trials may be compromised. The extent to which non-inferiority may be falsely declared also depends on the proportion of the trial participants who are assessed for TB disease, which is usually based on symptom screening. For a given diagnostic specificity, the number of false-positive diagnoses will increase with the proportion of trial participants who are tested for the disease endpoint, as will the probability of falsely declaring non-inferiority.

Vaccine non-inferiority trials using a marker of infection as the primary outcome must be designed and interpreted accounting for the precision of the tools used to measure infection; otherwise, they risk declaring non-inferiority by default. Until there is a test for TB infection with higher specificity, the use of the non-inferiority trial design for TB vaccine prevention of infection outcomes is not recommended.

## Data Availability

The simulated data for this study are available through the GitHub link given in the methods section.
